# Editorial: Research Collaboration and Networks: Characteristics, Evolution and Trends

**DOI:** 10.3389/frma.2021.690986

**Published:** 2021-05-07

**Authors:** Isola Ajiferuke, Maria Cláudia Cabrini Grácio, Siluo Yang

**Affiliations:** ^1^Faculty of Information and Media Studies, Western University, London, ON, Canada; ^2^São Paulo State University (UNESP), São Paulo, Brazil; ^3^School of Information Management, Wuhan University, Wuhan, China

**Keywords:** scientific collaboration, research networks, collaboration characteristics, research collaboration methodology, collaboration trends

## Introduction

In this age of big science, collaboration is increasingly playing an important role in implementing major scientific breakthroughs, solving complex scientific problems, promoting cultural understanding, improving research efficiency, achieving research excellence and knowledge innovation, and sharing scientific and technological resources, etc. (Andrade et al., 2009). Generally speaking, the research network is the manifestation of scientific cooperation. As a popular Research Topic, works on scientific collaboration and network have focused on a variety of terminologies and methods, diverse disciplines and views, different stages and levels, and various factors and challenges (Newman, 2001; Sonnenwald, 2007).

This Research Topic aims to discuss novel approaches and new insights to uncover the characteristics of research collaboration networks and to bring new perspectives to understand the nature and trends of scientific collaboration.

## Papers in This Research Topic

The six papers published in this Research Topic were all reviewed by two independent reviewers.

In the paper “Ontology-Based Graphs of Research Communities: A Tool for Understanding Threat Reduction Networks,” Ambrosiano et al. propose a method for storing, manipulating, and visualizing complex multidimensional research networks using an ontology-based graph database. The authors used the U.S. Biological Threat Reduction Program (BTRP) as a case study, and analyzed a series of seven BTRP genome sequencing training workshops, showing how the workshops created a growing network of participants and countries over time. The ontology-based approach, which involved capturing concept and relationship hierarchies, is applicable to other research communities or multidimensional social networks.

In the paper “Collaborative Patterns, Productivity, and Research Impact in the Careers of Star Researchers in a Japanese Semiconductor Company,” Maeki et al. demonstrate how long-term exposition and collaboration with other high-achieving researchers can play a significant role in determining a successful career in terms of productivity and impact. A collaborative network of 6,057 inventors and a subset of 15 star researchers in a Japanese semiconductor company over a long period of time was analyzed. Their results suggest that staying aligned in one research direction, long-term exposure to a diverse group of researchers, and early mentorship helped the researchers to attain their achievements.

In the paper “Then and Now, Mapping the 25 Year Evolution and Impact of North American Vascular Biology Organization Science Through Publications of its Founding and Current Members,” Galis et al. present general methods to study the evolution of a biomedical organization and its impact over time. Publications by members of the North American Vascular Biology Organization (NAVBO) over a 25-year period were collected and analyzed to understand how NAVBO fostered scientific collaborations and exchanges of expertise. UCSD Map of Science and Classification System was then used to show the evolution of scientific topics covered by NAVBO members.

In the paper “Collaborative Processes in Science and Literature: an In-Depth Look at the Cases of CERN and SIC,” Leogrande and Nicassio examine how the process of collaboration works in science and literature. For this purpose, the authors compared the collaboration process at the Center Européen pour la Recherche Nucléaire (CERN) on high-energy physics with the collaboration process used at Scrittura Industriale Collettiva (SIC) to produce a Great Open Novel, a collective book written by hundreds of people. The authors conclude that, no matter the field or the number of people it involves, a process of collaboration needs a structure to organize its plurality; this structure implies a path to follow, a task division, a constant coordination and control of participants' contributions.

In the paper “Scientific Collaboration at National Institute of the Atlantic Forest (Brazil) on Scopus Database: Analysis of Institutional Domain,” Freitas and Rosas introduce co-authorship analysis as a method in determining institutional domain. Using 138 articles published by 41 researchers at the National Institute of the Atlantic Forest, the authors demonstrate the potential of co-authorship analysis for the creation of a scientific and collaborative identity in an institutional domain.

In the paper “Mapping Collaborations and Partnerships in SDG Research,” Payumo et al. examine research output and collaboration supporting the United Nations Sustainable Development Goals (SDGs). The authors employed two additional lenses of collaboration, repeat collaboration and collaboration time point, to quantify and visualize co-authorship data sourced from Microsoft Academic Graph. The study's results show an increased collaboration rate over time but the majority of collaborations in SDG-related research only happened once. Hence, the authors identified core institutions that could help influence more consistent collaboration and sustain or grow the SDG-related research network.

## Conclusion

The articles published in this Research Topic have contributed to research collaboration methodology by: demonstrating the use of ontology-based approach to analyze research communities; adopting co-authorship analysis as a method in determining institutional domain; and employing two additional lenses of collaboration, repeat collaboration and collaboration time point, to quantify and visualize co-authorship data. In terms of practice, it has been shown that: by staying aligned in one research direction and getting exposed to a diverse group of researchers, a researcher can achieve success in terms of productivity and impact; and that no matter the field or the number of people it involves, a process of collaboration needs a structure to organize its plurality.

We hope that in the future, especially with the development of big data and artificial intelligence, more consequential research on theoretical frameworks, methods, and applications will be conducted in the area of scientific collaboration and networks.

## Author Contributions

All authors listed have made a substantial, direct and intellectual contribution to the work, and approved it for publication.

## Conflict of Interest

The authors declare that the research was conducted in the absence of any commercial or financial relationships that could be construed as a potential conflict of interest.
